# Stroke prevention in atrial fibrillation changes after dabigatran availability in China: The GLORIA‐AF registry

**DOI:** 10.1002/joa3.12321

**Published:** 2020-03-10

**Authors:** Changsheng Ma, Lionel Riou França, Shihai Lu, Hans‐Christoph Diener, Sergio J. Dubner, Jonathan L. Halperin, Qiang Li, Miney Paquette, Christine Teutsch, Menno V. Huisman, Gregory Y. H. Lip, Kenneth J. Rothman, the GLORIA‐AF Investigators

**Affiliations:** ^1^ Cardiology Department Atrial Fibrillation Center Beijing Anzhen Hospital Capital Medical University Beijing China; ^2^ Sanofi‐Aventis Recherche et Développement Chilly‐Mazarin France; ^3^ Biostatistics and Data Sciences Department Boehringer Ingelheim Pharmaceuticals, Inc. Ridgefield CT USA; ^4^ Department of Neurology University of Duisburg‐Essen Essen Germany; ^5^ Clínica y Maternidad Suizo Argentina Buenos Aires Argentina; ^6^ Icahn School of Medicine at Mount Sinai New York NY USA; ^7^ Boehringer Ingelheim (China) Investment Co. Ltd. Beijing China; ^8^ Department of Medicine Boehringer Ingelheim Burlington ON Canada; ^9^ Department of Thrombosis and Hemostasis Leiden University Medical Center Leiden the Netherlands; ^10^ Liverpool Centre for Cardiovascular Science University of Liverpool and Liverpool Heart & Chest Hospital Liverpool UK; ^11^ Aalborg Thrombosis Research Unit Department of Clinical Medicine Aalborg University Aalborg Denmark; ^12^ RTI Health Solutions Research Triangle Institute Research Triangle Park NC USA

**Keywords:** anticoagulants, antiplatelet agents, atrial fibrillation, delivery of health care, stroke

## Abstract

**Background:**

Until the approval of dabigatran etexilate, treatment choices for stroke prevention in patients with atrial fibrillation (AF) were vitamin K antagonists (VKAs) or antiplatelet drugs. This analysis explored whether availability of non‐vitamin K antagonist oral anticoagulants post‐dabigatran approval was associated with changing treatment patterns in China.

**Methods:**

Global Registry on Long‐Term Oral Antithrombotic Treatment in Patients with Atrial Fibrillation (GLORIA‐AF) collected data on antithrombotic therapy choices for patients with newly diagnosed nonvalvular AF at risk for stroke. In China, enrollment in phase 1 (before dabigatran approval) and phase 2 (after dabigatran approval) occurred from 2011 to 2013 and 2013 to 2014, respectively. Analyses were restricted to sites within China that contributed patients to both phases. The weighted average of the site‐specific results was estimated for standardization. Sensitivity analyses used multiple regression.

**Results:**

Thirteen sites participated in both phase 1 (419 patients) and phase 2 (276 patients), 76.1% and 16.0% were known to be at high risk for stroke (CHA_2_DS_2_‐VASc ≥2) and bleeding (HAS‐BLED ≥3); 55.5% were male. In phase 1, 16.7%, 61.6%, and 21.7% of patients were prescribed oral anticoagulants (OACs), antiplatelet agents, and no treatment, respectively. Respective proportions were 26.4%, 40.6%, and 33.0% in phase 2. The absolute increase in the site‐standardized proportion of patients prescribed OACs after dabigatran availability was 9.9% (95% confidence interval [CI]: 3.7%‐16.0%). There was a standardized 17.3% (95% CI: −24.3% to −10.4%) absolute decrease in antiplatelet agent use.

**Conclusions:**

There was an increase in OAC and decrease in antiplatelet agent prescription since dabigatran availability in China. However, a large proportion of AF patients at risk for stroke remained untreated.

## Graphical abstract

Using the Global Registry on Long‐Term Oral Antithrombotic Treatment in Patients with Atrial Fibrillation registry, the evolution of oral anticoagulation use in patients at risk for stroke was explored in China before and after the availability of the first non‐vitamin K antagonist oral anticoagulant. There was an increase in oral anticoagulation use coupled with a decrease in the use of antiplatelet agents alone; however a vast proportion of patients remained untreated.

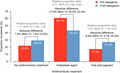

## 1 INTRODUCTION

Oral anticoagulant (OAC) therapy prevents ischemic stroke and reduces mortality in atrial fibrillation (AF) patients.^1^, ^2^ OAC therapy is underused in Asian countries, including China.^3^, ^4^ A 2008‐2009 report from the China National Stroke Registry demonstrated that 14% of nonvalvular AF patients with a history of ischemic stroke or transient ischemic attack were treated with a vitamin K antagonist (VKA) and 61% with antiplatelet agents without VKAs, while 25% received no antithrombotic therapy.^5^ Suggested explanations are a perceived higher baseline risk of anticoagulant‐induced intracerebral hemorrhage among Chinese patients,^6^, ^7^ a belief there is a low risk of ischemic stroke among Chinese AF patients^8^, ^9^ and the potential for herb‐drug interactions due to the high prevalence of herbal consumption.^10^ Warfarin interacts with many drugs and food ingredients, which may pose significant challenges in administration and monitoring.^9^ In addition, it has been stated that many patients in China lack access to good laboratory control for VKA therapy and therefore cannot be prescribed VKAs.^11^, ^12^

Non‐vitamin K antagonist oral anticoagulants (NOACs) have been suggested to be preferentially indicated over warfarin for Asian patients.^11^, ^13^ Dabigatran etexilate was the first NOAC on the market. It was approved by the China Food and Drug Administration on 22 February 2013 for stroke prevention in AF patients and has been available in Hong Kong since 14 July 2011 (rivaroxaban, another NOAC, was approved on 4 May 2015). New medicines must be registered with the China Food and Drug Administration prior to selection for inclusion within China's two major health insurance programs (rural and urban). In practice, dabigatran was included in the National Drug Reimbursement List in 2017, the first update of the list since 2009.

Several guidelines and consensus documents have been published in China to optimize anticoagulation therapy for stroke prevention in patients with AF^14^, ^15^ and, following the introduction of NOACs, more educational programs have been conducted.^3^

Given the obstacles to VKA use in China, the introduction of dabigatran (along with the contemporaneous expert consensus and educational programs) can be expected to have increased the proportion of patients prescribed OACs for stroke prevention in AF. The objective of this analysis was to explore whether availability of NOACs was associated with changing treatment patterns in China by comparing the proportion of treated patients between pre‐ and post‐dabigatran approval periods.

## 2 METHODS

### 2.1 Study design, participants, and setting

The design of the Global Registry on Long‐Term Oral Antithrombotic Treatment in Patients with Atrial Fibrillation (GLORIA‐AF) program has been reported previously.^16^ GLORIA‐AF is an ongoing disease registry of newly diagnosed nonvalvular AF patients at risk for stroke, run in three separate phases (clinical trial identifier: NCT01468701). In phase 1, before approval of dabigatran, information was collected before NOACs became available on the market. This initial phase used a cross‐sectional approach, with no data collected beyond the initial visit. From May 2011 (the date of the first patient in) until January 2013 (last patient in), 1063 eligible patients were enrolled, including 713 (67.1%) in China. VKA prescriptions were lower in China than other participating regions.^12^ Phase 2 started when dabigatran was approved in each respective country. During this phase, baseline patient characteristics were collected regardless of OAC prescription, and patients initiating dabigatran were followed for up to 2 years. The OAC prescription rate was low in China.^4^ Phase 3, which is ongoing, was initiated when characteristics of patients prescribed dabigatran and VKAs overlapped sufficiently to allow comparative analyses; a 3‐year follow‐up is ongoing for all included patients.

GLORIA‐AF sites were selected on the basis of confirmation that they diagnosed and followed up patients with AF, with no prerequisite for previous research experience. For a site to be eligible for inclusion, both dabigatran and VKAs must have been available or their availability must have been foreseen. Sources for site identification included professional directories, referrals from selected investigators and national coordinators, and sites that had previously worked with the study sponsor. Different health‐care settings (general practices, specialist offices, community hospitals, university hospitals, etc) were included worldwide. Physicians were encouraged to consecutively enroll unselected consenting patients meeting the eligibility criteria. GLORIA‐AF was performed in accordance with the provisions of the Declaration of Helsinki, and the protocol and procedures were approved by the European Medicines Agency, as well as relevant institutional review boards and ethics committees where required. All patients provided written informed consent before entering the registry. Patients were cared for by routine standard practice and were not required to be prescribed any specific treatment. In both phases 1 and 2, eligible patients were aged ≥18 years with newly diagnosed (<3 months) nonvalvular AF at risk for stroke (CHA_2_DS_2_‐VASc score ≥1). Patients with mechanical heart valves, prior VKA therapy for >60 days, life expectancy ≤1 year at enrollment, medical conditions other than AF for which long‐term use of VKAs is indicated, and AF due to a generally reversible cause were excluded.

This analysis focuses on eligible patients from phases 1 and 2 of GLORIA‐AF, enrolled by sites from mainland China that had patients included in both phases. Patients were enrolled in phase 1 from December 2011 to January 2013 and in phase 2 from September 2013 to June 2014. Database lock for the analysis was done in March 2015. In this exploratory analysis, no a priori sample size calculation was performed, and all eligible patients were included.

### 2.2 Variables

Baseline characteristics and antithrombotic treatment strategy were collected using electronic case report forms at the time of the enrollment (baseline) visit. The main outcome for this analysis was prescription of an OAC, excluding patients prescribed multiple OACs, but including patients prescribed an OAC in association with an antiplatelet drug. Prescription of an antiplatelet drug alone was a secondary outcome. Antithrombotic treatment strategy was defined based on antithrombotic treatments prescribed for long‐term use at the time of the baseline visit, or any antithrombotic treatments that the patient was already using at the time of the baseline visit and would continue using long term.

Baseline patient characteristics included demographic and clinical data, AF characteristics, medical history, and selected concomitant treatments. Labile international normalized ratio was not collected at baseline; a modified hypertension, abnormal renal function, abnormal liver function, stroke (prior), bleeding (prior), labile international normalized ratio, elderly (age >65 years), prior alcohol or drug usage history, medication usage predisposing to bleeding (antiplatelet agents, nonsteroidal anti‐inflammatory drugs) (HAS‐BLED) score not considering labile international normalized ratio was used.

Potential confounders in the association between the phase of the study and antithrombotic prescriptions considered were as follows: risk scores for stroke (CHA_2_DS_2_‐VASc) and bleeding (modified HAS‐BLED), age, sex, body mass index, AF characteristics (categorization as asymptomatic, minimally symptomatic, or symptomatic; and type as permanent, persistent, or paroxysmal AF), history of thromboembolic disease (stroke), cardiovascular history (myocardial infarction, coronary artery disease, congestive heart failure/left ventricular dysfunction, hypertension), history of bleeding events, other medical history (diabetes mellitus, chronic gastrointestinal disease), enrollment site, and month of enrollment.

### 2.3 Statistical methods

The objective was to assess the changes in antithrombotic treatment patterns after dabigatran availability, which involved comparing patterns during phases 1 and 2 of the GLORIA‐AF registry program.

To avoid bias due to different sites participating in the study phases, analyses were restricted to sites from mainland China (the main country enrolling patients in phase 1) enrolling patients in both phases 1 and 2.

As site participation may have differed between phases 1 and 2 of the study, and sites may have differed in their antithrombotic prescription patterns, the main analysis involved within‐site comparisons with standardization across sites.^17^ Standardization involved taking a weighted average of the site‐specific results, weighting by the total number of patients enrolled by each site during both phases.

Patient characteristics were described according to phase. We conducted a sensitivity analysis using log‐binomial regression^18^ to account for phase, site, and characteristics imbalanced between phases 1 and 2. Log‐binomial regression provides probability (in this case, antithrombotic treatment prescription) and probability ratios (in this case, the ratio between phase 2 and phase 1). Imbalanced characteristics were defined as any variable for which the absolute value of the standardized difference^19^ between phase 1 and phase 2 was higher than 10%. The standardized difference compared the difference in means in units of the pooled standard deviation.

For multiple regression, missing values were treated as an absence of the considered risk factor; standardized differences were also assessed after single imputation for missing values.

To avoid confusion between the use of “standardized” for imbalance diagnosis (standardized difference) and of “standardization” as our main analysis approach controlling for site effects, “site‐standardized” will be used in the remainder of the article to describe the latter.

The proportion of OAC use during baseline was also described according to the month of baseline visit for both phases 1 and 2 to assess underlying time trends. In addition, a log‐binomial model estimating the association between time and OAC use was fitted to the subset of patients enrolled in phase 1, adjusting for site and patient characteristics found to be imbalanced between study phases. A potential phase 2 trend was not explored by multivariable modeling, since the focus was on assessing the extent to which the change in OAC use observed in phase 2 could be explained by a prescription trend before dabigatran availability. Inclusion of both phase and time as predictors in a multiple regression was not reasonable because of high multicollinearity between time and phase.

## 3 RESULTS

### 3.1 Participants

**FIGURE 1:**
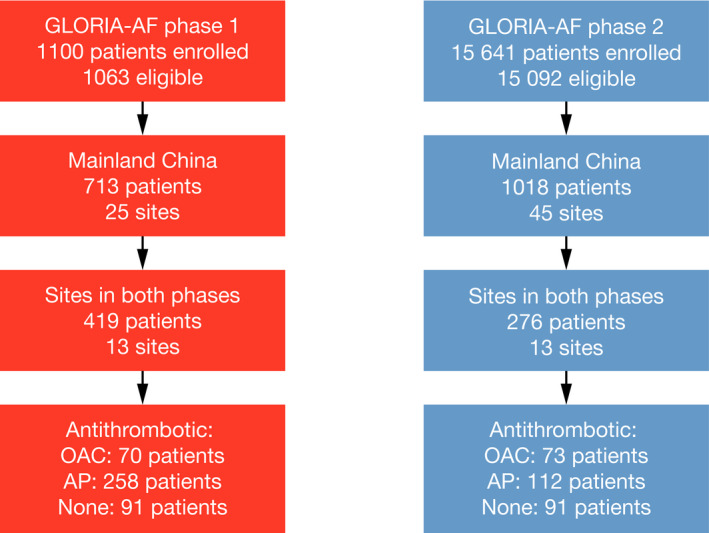
Flow chart of patients considered for the analysis. AP, antiplatelet agent; OAC, oral anticoagulant

Treatments prescribed and baseline characteristics of overall patients in phase 1^12^ and phase 2^4^, ^20^ are described elsewhere. In mainland China, 25 sites enrolled 713 eligible patients in phase 1 and 45 sites enrolled 1018 eligible patients in phase 2. Thirteen sites contributed to both phase 1 (419 patients enrolled) and phase 2 (276 patients enrolled) (Figure 1).

Sites participating only in phase 1 had fewer eligible patients per site (24.5 ± 12.5 on average vs 32.2 ± 11.6 patients per site in phase 1 for sites contributing to both phases). The average number of eligible phase 2 patients per site was 23.2 ± 30.2 for sites participating in phase 2 only and 21.2 ± 11.8 for sites participating in both phases. The rate of OAC prescription in phase 1 was higher for sites participating in phase 1 only (26% vs 17% for sites participating in both phases), possibly due to patients having a lower HAS‐BLED score (1.2 ± 0.98 vs 1.6 ± 0.95, respectively). In phase 2, the converse was observed, with a lower proportion of OAC prescriptions for sites participating in phase 2 only compared with those participating in both phases (19% vs 26%, respectively), despite similar HAS‐BLED scores (1.2 ± 0.88 vs 1.2 ± 0.95, respectively).

The analyses restricted to sites contributing to both phases involved 695 patients, enrolled from 13 sites located in Beijing (n = 5, 41% of patients); Shanghai (n = 3, 21%); Guangzhou (n = 2, 10%); Nanjing, Hangzhou, and Changsha (one site each; 11%, 10%, and 6%, respectively). In both phases combined, most patients were enrolled in university hospitals (489 patients, 70%), with community hospitals (119, 17%), and specialist offices (87, 13%) being less common. Most patients were treated by a cardiologist (681, 98%).

### 3.2 Patient characteristics

There were signs of imbalance (standardized difference >10%) in some patient characteristics between phase 1 (pre‐dabigatran approval) and phase 2: category of AF (65% of patients in phase 1 and 44% in phase 2 were symptomatic; standardized difference: +43%), known high‐bleeding risk (modified—not considering labile international normalized ratio—HAS‐BLED ≥3), body mass index, known prior bleed, congestive heart failure, gastrointestinal disease, and prior myocardial infarction (Table 1).

**TABLE 1:** Patient characteristics according to study phase

	Phase 1 (pre‐dabigatran) n = 419	Phase 2 (post‐dabigatran) n = 276	Standardized difference
Demographics			
Age, mean (SD)	68.1 (11.75)	67.2 (11.71)	0.08
Female, n (%)	185 (44.2)	124 (44.9)	−0.02
BMI, mean (SD)	23.8 (3.58)	24.5 (3.75)	−0.19
Risk scores, n (%)			
CHA_2_DS_2_‐VASc ≥2	318 (75.9)	211 (76.4)	−0.01
Modified HAS‐BLED^a^ ≥3	63 (15.0)	24 (8.7)	0.20
AF characteristics, n (%)			
Type of AF			
Paroxysmal	276 (65.9)	182 (65.9)	−0.00
Persistent	139 (33.2)	90 (32.6)	0.01
Permanent	4 (1.0)	4 (1.4)	−0.05
Category of AF			
Asymptomatic	66 (15.8)	60 (21.7)	−0.15
Minimally symptomatic	81 (19.3)	95 (34.4)	−0.35
Symptomatic	272 (64.9)	121 (43.8)	0.43
Medical history, n (%)			
Stroke	43 (10.3)	36 (13.0)	−0.09
Bleeding	23 (5.5)	7 (2.5)	0.15
Myocardial infarction	27 (6.4)	27 (9.8)	−0.12
Coronary artery disease	115 (27.4)	75 (27.2)	0.01
Congestive heart failure^b^	86 (20.5)	73 (26.4)	−0.14
Hypertension	300 (71.6)	192 (69.6)	0.04
Diabetes mellitus	83 (19.8)	57 (20.7)	−0.02
Gastrointestinal disease	40 (9.5)	17 (6.2)	0.13

Abbreviations: AF, atrial fibrillation; BMI, body mass index; HAS‐BLED, hypertension, abnormal renal function, abnormal liver function, stroke (prior), bleeding (prior), labile international normalized ratio, elderly (age >65 y), prior alcohol or drug usage history, medication usage predisposing to bleeding (antiplatelet agents, nonsteroidal anti‐inflammatory drugs); SD, standard deviation.

^a^ Patients with missing HAS‐BLED (n = 13) were considered at low risk.

^b^ Congestive heart failure/left ventricular dysfunction.

### 3.3 Antithrombotic treatment strategies

**FIGURE 2:**
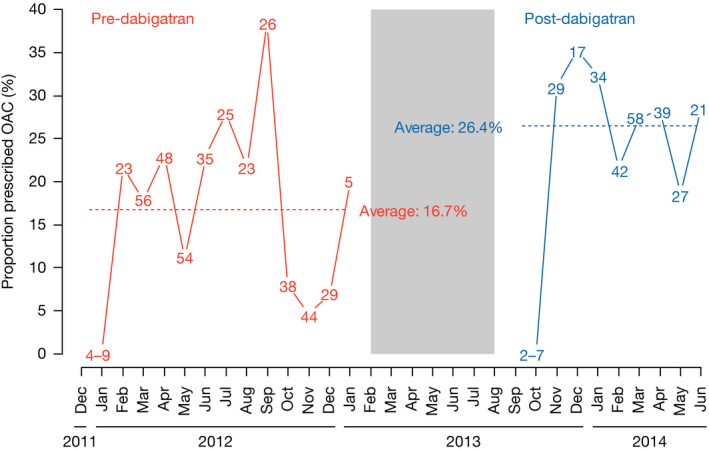
Enrolled patient numbers and crude proportion of OAC use over time. Numbers in the lines correspond to the number of patients enrolled in each month. OAC, oral anticoagulant

Figure 2 presents the proportion of patients prescribed OACs per month. Sites were not active in China between February and August 2013 (transition period between phases 1 and 2). In phase 1, 17% of patients were prescribed OACs; this proportion varied from 0% to 41% depending on the site. In phase 2, the proportion was 26%, varying from 0% to 67%. In addition, site contributions were different according to phase; at the extremes, one site contributed 12% of phase 1 patients vs 7% of phase 2 patients, and another site contributed 8% vs 15%, respectively. Enrollment site was therefore associated with both phase and treatment prescribed.

**FIGURE 3:**
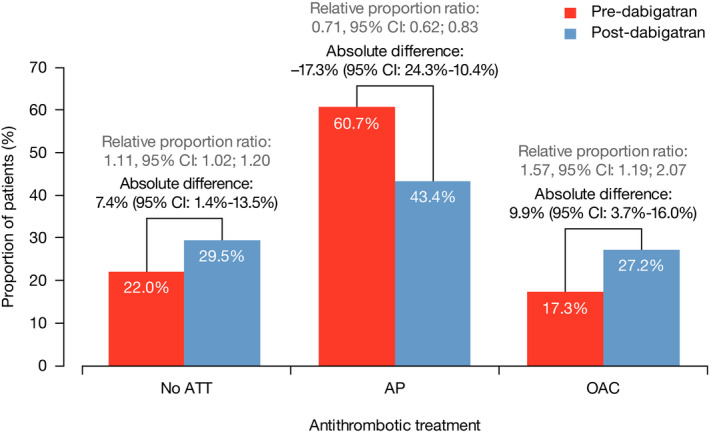
Site‐standardized antithrombotic treatment prescription probability differences and probability ratios from phase 1 to phase 2. AP, antiplatelet agent; ATT, antithrombotic treatment; CI, confidence interval; OAC, oral anticoagulant

Table 2 and Figure 3 present the crude and site‐standardized proportions of treatments prescribed, along with site‐standardized estimates of the difference in proportions between phase 2 and phase 1 as well as the ratio of proportions. From phase 1 to phase 2, standardizing by site, the proportion of patients receiving OACs increased by 10 percentage points (95% confidence interval [CI]: 4‐16), corresponding to a relative increase of (0.272/0.173 − 1) = 57% (95% CI: 19%‐107%).

**TABLE 2:** Antithrombotic treatment and study phase

Treatment	Crude N (%)	
	Phase 1 (pre‐dabigatran) n = 419	Phase 2 (post‐dabigatran) n = 276
OAC	70 (16.7)	73 (26.4)
Of which NOAC	—	8 (2.3)
Antiplatelet agent	258 (61.6)	112 (40.6)
None	91 (21.7)	91 (33.0)

Abbreviations: OAC, oral anticoagulant (±antiplatelet drug); NOAC, non‐vitamin K antagonist oral anticoagulant (±antiplatelet drug).

The increase in OAC use came with a decrease (−17%) in antiplatelet drug use alone but also an increase (+7%) in the proportion of untreated patients.

By definition, no NOAC was available in phase 1 (before availability of dabigatran) and all OAC use corresponded to VKA use. In phase 2, eight patients were prescribed NOACs, constituting 2% of all phase 2 patients, in addition to 24% prescribed VKAs.

### 3.4 Sensitivity analyses

When controlling for site, body mass index, bleeding risk (modified HAS‐BLED), category of AF, history of bleeding, myocardial infarction, congestive heart failure, and chronic gastrointestinal disease in a multiple log‐binomial regression model, OAC use increased by 47% in phase 2 (95% CI: 10%‐97%) (Table 3). This increase was 57% (95% CI: 19%‐107%) in the main analysis standardizing by site.

**TABLE 3:** Sensitivity analyses: probability ratios and 95% CIs from the log‐binomial regression models

Variable	Phases 1 and 2 n = 695	Phase 1 Only n = 419
Phase (ref: phase 1)	1.47 (1.10‐1.97)	Not included
Time (continuous, months since December 2011)	Not included	0.94 (0.86‐1.01)
BMI <30 kg/m^2^ (ref: ≥30 kg/m^2^)	1.14 (0.70‐2.09)	0.46 (0.28‐1.36)
Modified HAS‐BLED^a^ ≥3 (ref: score <3)	0.76 (0.43‐1.20)	0.73 (0.32‐1.39)
Category of AF (ref: asymptomatic)		
Symptomatic	0.62 (0.44‐0.90)	0.82 (0.48‐1.46)
Minimally symptomatic	0.69 (0.48‐1.01)	0.53 (0.26‐1.07)
History of bleeding^a^	1.20 (0.57‐2.05)	1.19 (0.48‐2.24)
Myocardial infarction	0.21 (0.05‐0.57)	0.68 (0.13‐1.93)
Congestive heart failure	0.82 (0.52‐1.23)	0.82 (0.42‐1.51)
Gastrointestinal disease	0.43 (0.15‐0.91)	0.38 (0.08‐1.03)

Note

Intercept and the 12 coefficients associated with the 13 sites are not shown.

Abbreviations: AF, atrial fibrillation; BMI, body mass index; CI, confidence interval; HAS‐BLED, hypertension, abnormal renal function, abnormal liver function, stroke (prior), bleeding (prior), labile international normalized ratio, elderly (age >65 y), prior alcohol or drug usage history, medication usage predisposing to bleeding (antiplatelet agents, nonsteroidal anti‐inflammatory drugs).

^a^ Patients with missing prior bleed (n = 1) or HAS‐BLED (n = 13) were considered at low risk.

Differences between phase 2 and phase 1 of the study may be related to a temporal trend that predates dabigatran availability. In Figure 2, no clear trend is apparent during either phase 1 or 2. A time trend could be masked by variations in patient characteristics over time. When predicting OAC use over time during phase 1, controlling for site, body mass index, bleeding risk (modified HAS‐BLED), category of AF, history of bleeding, myocardial infarction, congestive heart failure, and chronic gastrointestinal disease in a multiple log‐binomial regression model (Table 3, column 3), there was, however, no indication of a linear increase in OAC prescriptions over time (probability ratio of OAC prescription from one month to the next: 0.94, 95% CI: 0.86‐1.01).

## 4 DISCUSSION

The principal study findings are as follows: (a) OAC use increased in China after NOACs became available; (b) this increase is only partly driven by an increase in NOAC use; and (c) in parallel, antiplatelet agent use decreased.

When considering available guidelines, OACs,^21^ and particularly NOACs, are becoming the standard of care for stroke prevention in AF worldwide; they are already recommended in preference to VKAs in various guidelines.^2^, ^22^ The 2016 Chinese expert consensus on the management of AF in the elderly population recommends using NOACs to treat older AF patients (≥65 years), especially patients who cannot tolerate warfarin, patients who had bleeding after warfarin treatment, or those who have unstable international normalized ratio.^23^

In accordance with this trend, analyses from GLORIA‐AF after dabigatran availability (phase 2 of the registry program) have shown that uptake of NOACs was more prevalent than uptake of VKAs, except in Asia, where 28% of patients received VKAs and 28% NOACs.^20^ Country‐specific analyses^4^ indicate that oral anticoagulation use in mainland China is even lower than in other Asian locations, with 15% of patients prescribed VKAs and 6% a NOAC.

Low NOAC uptake may be related to availability status in China: while dabigatran has been available in China since February 2013, it was listed in the National Drug Reimbursement List in 2017, after the GLORIA‐AF phase 2 study period. Another explanation may be that, since the enrollment period in phase 2 was relatively short (10 months) in China (all locations combined, enrollment in phase 2 extended from December 2011 to December 2014), physicians had insufficient time to adapt to the new treatment availabilities.

Our analyses are consistent with an increase of OAC use and a decrease in antiplatelet drug use after dabigatran availability. Considering that after NOACs became available, guidelines and consensus on OAC treatment optimization have been published and more educational programs have been conducted,^3^ this trend is perhaps not surprising. Other studies in the United States,^24^ Denmark,^25^ Australia,^26^ and Korea^27^ have reported similar trends, although some US studies also reported no increase in OAC prescriptions overall, with an increase in NOAC uptake being compensated for by a decrease in VKA use.^28^

Our observations are concordant with an analysis based on the Chinese Atrial Fibrillation Registry, involving 20 tertiary and 12 nontertiary hospitals in Beijing, which reveals considerable variability in OAC use across sites. The Chinese Atrial Fibrillation Registry also reported an increase in OAC use over time, especially in the period following dabigatran availability. In patients with CHA_2_DS_2_‐VASc ≥2, OAC use increased from 30.2% to 38.1% from August 2011 to August 2013, and to 57.7% by August 2014.^3^

In line with other registries, this analysis from GLORIA‐AF indicates that OAC use increased in China after dabigatran approval. Nonetheless, although all patients in the GLORIA‐AF registry had a CHA_2_DS_2_‐VASc score of ≥1, and 76% in this analysis had a CHA_2_DS_2_‐VASc score ≥2, many patients were treated with antiplatelet drugs alone (40.6% after dabigatran approval) or were not treated (33.0%). In this analysis, the increase in OAC use after dabigatran approval (+10%) was only partially explained by NOAC prescriptions alone (+2%). It is possible that, in a setting where NOACs were not covered by medical insurance, economic factors may have had a greater influence on what was prescribed. Nonetheless, NOAC approval may have had an indirect role, triggering guideline updates and increasing the awareness of optimal management of stroke prevention in AF.

### 4.1 Limitations

Some limitations should be considered. The number of patients available for the analyses was modest, limiting our ability to adjust for confounders and identify time trends. As phase 2 of the study started when dabigatran was approved in each respective country, the enrollment period in phase 2 was relatively short (10 months) in China where dabigatran was approved in February 2013 (compared with December 2011 to December 2014 for all locations combined). This may have limited the number of patients enrolled in China. In addition, analyses were restricted to mainland China. As such, our results illustrate the changing pattern of use of OACs and NOACs under the specific circumstances prevailing in China. Restriction to a single country has the advantage of controlling more effectively for geographic and cultural differences. In contrast, the findings may not be applicable to other Asian countries, especially those where reimbursement was possible shortly after NOAC approval. Furthermore, the 13 sites included in the present analysis may not be representative of all Chinese patients. The majority of patients were enrolled by university hospitals, which may have been quicker to change prescription behaviors. The change in the whole country may have been less marked. However, the trends observed in this analysis from phases 1 and 2 of the GLORIA‐AF registry program, adjusting for enrolling sites, are robust in our sensitivity analyses, consistent with other reported evidence from China^5^ and in line with expected evolutions in antithrombotic use after the introduction of NOACs and guideline updates.

## 5 CONCLUSION

The patterns observed in patients enrolled in GLORIA‐AF in mainland China are consistent with an increase in the proportion of patients prescribed OACs since dabigatran became available. In parallel, the rate of antiplatelet prescriptions showed a decrease following dabigatran availability and is in line with published guidelines. Despite the increase in oral anticoagulation use since dabigatran availability, a large proportion of patients remain untreated, or treated with antiplatelet only.

## CONFLICT OF INTEREST

This registry is registered in ClinicalTrials.gov with clinical trial identifier NCT01468701. The protocol for this research project has been approved by a suitably constituted Ethics Committee of the institutions, where required, and it conforms to the provisions of the Declaration of Helsinki. Beijing Anzhen Hospital, Capital Medical University, Beijing, China, institutional review board approval 11 July 2013. CM has received honoraria for presentations as well as research grants from Bristol‐Myers Squibb, Boehringer Ingelheim, Bayer HealthCare, Pfizer, AstraZeneca, and Johnson & Johnson. LRF was an employee of Boehringer Ingelheim at the time of manuscript writing and is now employed by Sanofi‐Aventis. MVH has received honoraria for presentations and research grants from Boehringer Ingelheim, Bayer HealthCare, Pfizer, GlaxoSmithKline, and Actelion Pharmaceuticals. H‐CD has received honoraria for participation in clinical trials, contribution to advisory boards or oral presentations from Abbott, Allergan, AstraZeneca, Bayer Vital, Bristol‐Myers Squibb, Boehringer Ingelheim, CoAxia, Corimmun, Covidien, Daiichi‐Sankyo, D‐Pharm, Fresenius, GlaxoSmithKline, Janssen‐Cilag, Johnson & Johnson, Knoll, Lilly, MSD, Medtronic, MindFrame, Neurobiological Technologies, Novartis, Novo‐Nordisk, Paion, Parke‐Davis, Pfizer, Sanofi‐Aventis, Schering‐Plough, Servier, Solvay, St. Jude, Synagis, Talecris, ThromboGenics, WebMD Global, Wyeth and Yamanouchi. Financial support for research projects was provided by AstraZeneca, GlaxoSmithKline, Boehringer Ingelheim, Lundbeck, Novartis, Janssen‐Cilag, Sanofi‐Aventis, Synagis, and Talecris. The Department of Neurology at the University Duisburg‐Essen received research grants from the German Research Council (DFG), the German Ministry of Education and Research (BMBF), the European Union, the National Institutes of Health (NIH), the Bertelsmann Foundation, and the Heinz‐Nixdorf Foundation; H‐CD has no ownership interest and does not own stocks of any pharmaceutical company; within the past year he served as the editor of *Aktuelle Neurologie*, *Arzneimitteltherapie*, *Kopfschmerznews*, and *Stroke News*, as the co‐editor of *Cephalalgia*, and was on the editorial board of *Lancet Neurology*, *Stroke*, *European Neurology*, and *Cerebrovascular Disorders*; he chairs the Treatment Guidelines Committee of the German Society of Neurology and has contributed to the European Heart Rhythm Association (EHRA) and the European Society of Cardiology (ESC) guidelines for the treatment of atrial fibrillation. SJD has received consultancy fees for serving as a steering committee member for Boehringer Ingelheim. He also holds research grants from St Jude Medical. JLH has received consulting fees from Bayer HealthCare, Janssen‐Ortho‐McNeil and Pfizer for advisory activities involving the development of anticoagulant drugs. GYHL has been a consultant for Bayer/Janssen, Bristol‐Myers Squibb/Pfizer, Medtronic, Boehringer Ingelheim, Novartis, Verseon, and Daiichi‐Sankyo; and speaker for Bayer, Bristol‐Myers Squibb/Pfizer, Medtronic, Boehringer Ingelheim, and Daiichi‐Sankyo. He has not received any personal fees. KJR is an employee of RTI Health Solutions, an independent nonprofit research organization that does work for government agencies and pharmaceutical companies. SL, QL, MP, and CT are employees of Boehringer Ingelheim.
